# Linking differential radiation responses to glioma heterogeneity

**DOI:** 10.18632/oncotarget.1823

**Published:** 2014-03-12

**Authors:** Chao Ke, Katherine Tran, Yumay Chen, Anne T. Di Donato, Liping Yu, Yuanjie Hu, Mark E. Linskey, Ping H. Wang, Charles L. Limoli, Yi-Hong Zhou

**Affiliations:** ^1^ Neurological Surgery, University of California, Irvine, CA, USA; ^2^ Sun Yat-sen University Cancer Center; State Key Laboratory of Oncology in South China; Collaborative Innovation Center for Cancer Medicine, Guangzhou, China; ^3^ Radiation Oncology, University of California, Irvine, CA, USA; ^4^ Medicine, University of California, Irvine, CA, USA

**Keywords:** glioma, cellular heterogeneity, tumorigenicity, redox homeostasis, metabolic state, radiosensitivity

## Abstract

The phenotypic and genetic diversity that define tumor subpopulations within high-grade glioma can lead to therapeutic resistance and tumor recurrence. Given that cranial irradiation is a frontline treatment for malignant glioma, understanding how irradiation selectively effects different cellular subpopulations within these heterogeneous cancers should help identify interventions targeted to better combat this deadly disease. To analyze the radiation response of distinct glioma subpopulations, 2 glioma cells lines (U251, A172) were cultured under conditions that promoted either adherence or non-adherent spheroids. Past work has demonstrated that subpopulations derived from defined culture conditions exhibit differences in karyotype, proliferation, gene expression and tumorigenicity. Spheroid cultures from each of the glioma cell lines were found to be more radiosensitive, which was consistent with higher levels of oxidative stress and lower levels of both oxidative phosphorylation and glycolytic metabolism 1 week following irradiation. In contrast, radioresistant non-spheroid parental cultures showed increased glycolytic activity in response to irradiation, while oxidative phosphorylation was affected to a lesser extent. Overall these data suggest that prolonged radiation-induced oxidative stress can compromise the metabolic state of certain glioma subpopulations thereby altering their sensitivity to an important therapeutic intervention used routinely for the control of glioma.

## INTRODUCTION

Tumor heterogeneity at both primary and distant metastatic sites has been observed in most cancers at the time of clinical diagnosis [1, 2], where evidence of genetic clonal diversity has been linked to tumor progression [3]. The complex interplay between tumor subpopulations defines the evolution of many cancers [4], and drives the selective pressure for tumor growth and survival against the challenges of radio- and chemotherapy. Significant evidence has reinforced the importance of tumor heterogeneity, where specific subpopulations of cells exhibit distinct phenotypes that compliment one another for achieving enhanced tumorigenicity and treatment resistance [5-7]. Multiple complex interactions regulate the transition between different subpopulations within a given cancer, which under certain circumstances can be achieved through epigenetic and/or transcriptional alterations in breast cancer [8], or via chromosome mis-segregation [6] or re-gain of chromosome double minute in malignant glioma [7]. Inter and intra-tumoral heterogeneity are well known for glioblastoma multiforme (the highest grade of glioma), which are complicated due to diverse tumor cell origins shown in mouse models of glioma [9] and patient’s tumor specimens [10, 11]. Therefore, a more detailed understanding of the differential responses to irradiation by distinct subpopulations of glioma cells should provide information useful for improving the curability of gliomas and other deadly cancers.

Exposure to ionizing radiation to eradicate cancer elicits a stress response in mammalian cells known to involve oxidative stress [12-15]. Radiation-induced oxidative stress impacts several critical biologic parameters in both normal and neoplastic tissue including survival, proliferation, DNA damage and repair, mitochondrial function and stem cell fate [13-18]. For many solid tumors, radioresistant subpopulations of cancer stem cells (CSCs) have been identified that respond to changes in redox state to drive tumor growth [19]. For glioblastoma multiforme (GBM), the highest grade glioma, CSC isolated based on expression of CD133 were shown to be more radioresistant than non-CSC subpopulations not expressing CD133 [20]. Other groups have used the same and other markers to define glioma subpopulations with distinct *in vitro* and *in vivo* phenotypes correlating to various degrees of tumorigenicity [21-24]. Reliance on specific markers to define tumor subpopulations is subject to inherent limitations, and to avoid many of these caveats, we developed a culture methodology that optimized the isolation of distinct subpopulations from established glioma cell lines. We have shown that these subpopulations exhibit differences in karyotype, proliferation, gene expression and tumorigenicity in subcutaneous and intracranial xenograft models [6]. Here we report how radiation-induced changes in the redox and metabolic state of glioma subpopulations might alter their sensitivity to irradiation, thereby impacting radiocurability.

## RESULTS

### Characterizing and isolating distinct subcultures from heterogeneous glioma cell lines

**Figure 1:**
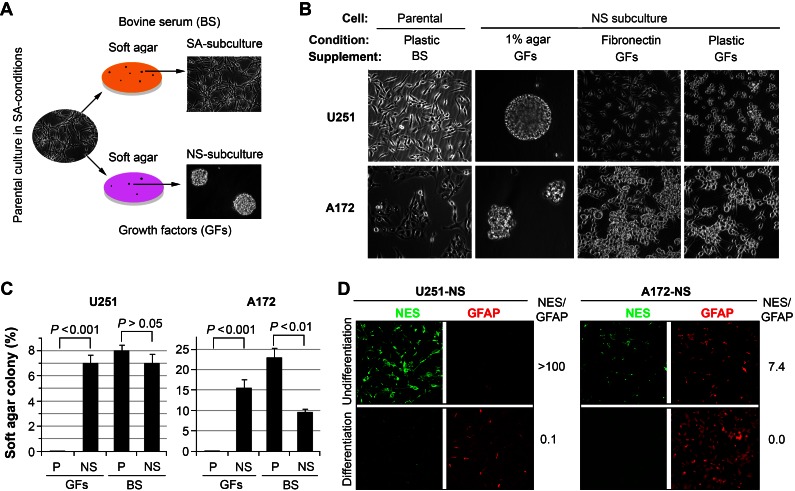
Differential growth of heterogeneous glioma cell lines A. Parental cultures were subcloned in soft agar to establish non-adherent spheroid (NS) and serum-adherent (SA) subpopulations that were subsequently maintained under defined yet distinct culture conditions. B. Phase contrast images showing the morphologic differences of cells grown under various culture conditions. C. Comparison of colony formation in soft agar containing bovine serum (BS) or growth factors (GFs) for parental (P) and NS subcultures of U251 and A172. Error bars correspond to SEM derived from 4 replicates. D. Differentiation of NS subcultures derived from U251 and A172 cell lines showing changes in nestin (NES, green) and GFAP (red) expression by immunohistochemistry. The ratio of fluorescent intensity (NES/GFAP) quantified by ImageJ is shown to the right of each paired panel.

Malignant gliomas are well known for being comprised of heterogeneous cell subpopulations. In our prior studies, we used fluorescence *in situ* hybridization (FISH) for analyzing copy number variation of chromosome 7 (Chr7) in various populations of primary gliomas and their derived cell lines [6]. Importantly, many of our prior and current findings of clonal heterogeneity were triggered by switching primary parental cells from serum adherent (SA) conditions to those promoting the growth of non-adherent neurospheres (NS) as summarized (Figure 1A). We found marked copy number variation in specific subpopulations of gliomas derived from single cells that were dependent on culture conditions. Established neurosphere cultures maintained in the presence of growth factors (EGF, bFGF) were also found to be more tumorigenic and harbor subpopulations with different variations in Chr7 [6]. In contrast to NS-subcultures, the karyotypes of cells cultured under SA conditions were similar to that of parental (P) cultures, which were originally established under the same SA culture conditions [6]. As with past studies, all glioma subpopulations were derived from subclones originally isolated from single colonies in soft agar. Thus, the clonal heterogeneity reported here developed during subsequent passage.

Interestingly, NS subcultures possess the capability to grow as multi-cellular spheroids in agar-coated dishes, or as monolayers in fibronectin-coated dishes. NS-subcultures derived from U251 and A172 exhibit differences in spheroid size and morphology and exhibit similar morphologic variations when grown as monolayer cultures compared to their parental cultures (Figure 1B). Both NS subcultures of U251 and A172 were able to form colonies in media containing either bovine serum (BS) or growth factors (GFs) (Figure 1C). In striking contrast, both parental cultures failed to grow in soft agar without providing BS, demonstrating the adaptive advantages imparted to NS subcultures (Figure 1C). NS cells derived from U251 exhibit significant multipotency, based on their ability to undergo glial and neuronal differentiation *in vitro* and endothelial trans-differentiation during the formation of intracranial tumors [6]. Separate NS cultures derived from A172 (A172-NS) and U251 (U251-NS) subjected to *in vitro* differentiation for 1 week showed similar reductions in the level of nestin (NES) while the expression of GFAP increased (Figure 1D). The ratio of fluorescent intensities (NES/GFAP, Figure 1D) indicates that NS subpopulations express relatively higher levels of nestin, which become undetectable after extended differentiation that promotes the expression of GFAP. As previously found in U251-NS cells [6], differentiation of A172-NS did not lead to detectable levels of neuronal markers (MAP2, β-Tubulin III). Very few cells in NS subcultures of U251 and A172 were stained by the CD133 antibody.

### Differential radiosensitivity of glioma subpopulations

**Figure 2:**
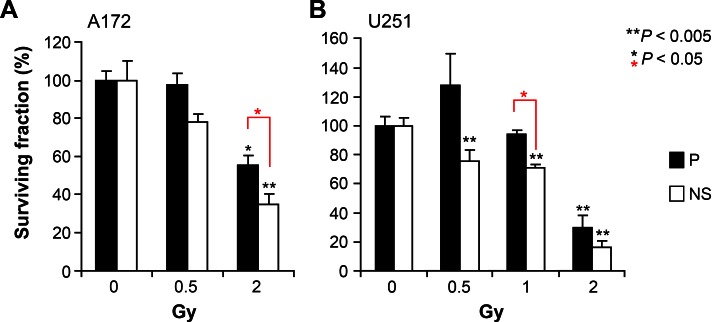
Differential radio sensitivity of glioma subpopulations Comparison of surviving fraction in response to acute single dose irradiation of parental and NS subcultures of A172 (A) and U251 (B). Error bars correspond to SEM derived from 4 replicates, based on the number of colonies formed in soft agar (1000 cells /per well).

Past and current characterization of glioma cell subpopulations has revealed phenotypic and genetic diversity, changes that could impact radiosensitivity by altering the capability of cells to engage in DNA damage responses [6, 25]. We used colony formation in soft agar to assess clonogenic survival under equivalent conditions for both parental and NS cells. Compared to unirradiated controls, a clinically relevant dose of 2 Gy significantly reduced survival (*P* < 0.05) in both parental and NS cells of A172 and U251 (Figure 2). Furthermore, NS cells showed greater sensitivity to radiation at doses of 2 Gy (A172, see Figure 2A) and 1 Gy (U251, see Figure 2B) and compared to their parental SA lines. Under most irradiation conditions, both parental and NS cells of U251 were more radiosensitive than those of A172.

### Radiation-induced oxidative stress and radiosensitivity

**Figure 3:**
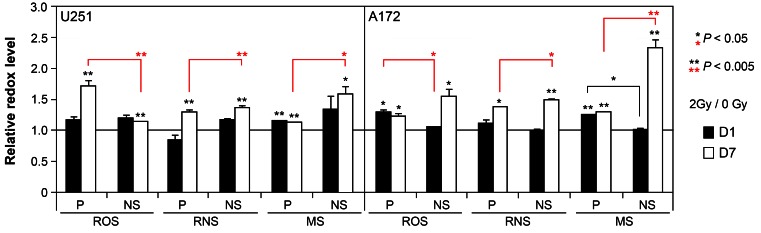
Acute and persistent oxidative stress in irradiated parental and NS subcultures of U251 and A172 cells Comparison of reactive oxygen (ROS), nitrogen (RNS) species and mitochondrial superoxide (MS) levels measured by fluorescence activated cell sorting of cells loaded with CMH_2_DCFDA, DAF, and MitoSOX, respectively, 1 and 7 days after irradiation (2 Gy). All values normalized to unirradiated controls set to unity. Error bars represent SEM derived from 4 replicates. Significance between unirradiated controls (asterisks above each bar) and between parental and NS subcultures (asterisks above brackets) are shown.

Past work from our lab has demonstrated that exposure to ionizing radiation elicits an acute and persistent oxidative stress in a range of multipotent neural stem and progenitor cells that can impact a variety of cell survival parameters. To determine the extent that similar (or dissimilar) redox changes occur in glioma subcultures, relative levels of reactive oxygen (ROS) and nitrogen (RNS) species were assessed using redox sensitive fluorogenic dyes at early (1 day), and protracted (7 days) times following exposure to a clinically relevant dose of 2 Gy. Cells subjected to irradiation and loaded with the dyes CM-H_2_DCFDA, DAF or Mitosox, were subjected to fluorescence activated cell sorting (FACS) to quantify fluorescent signals proportional to the intracellular level of ROS, RNS (nitric oxide) and mitochondrial superoxide (MS). While relatively modest increases in oxidative stress were observed 1 day following irradiation in subcultures derived from both cell lines, the levels of ROS, RNS and MS were elevated significantly 7 days after exposure compared to sham irradiated controls set to unity (Figure 3). Moreover, at this protracted post-irradiation interval, the levels of reactive species were generally higher for each NS subculture compared to their corresponding parental cultures (Figure 3). For each data set, significance compared to unirradiated controls is indicated (asterisks above each bar) along with significance between parental and NS subcultures (asterisks above brackets).

### Metabolic changes in glioma subcultures in response to irradiation

**Figure 4:**
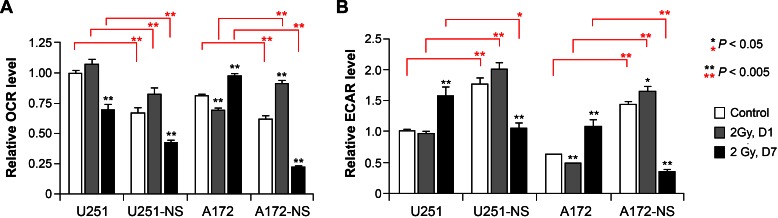
Cellular bioenergetics measured by flux analysis of irradiated parental and NS subcultures of U251 and A172 cells Comparison of oxygen consumption rate (OCR) and extracellular acidification rate (ECAR) using an extracellular flux analyzer before and after irradiation. To facilitate comparisons between cells treatments and measurements, the OCR and ECAR levels of U251 controls were set to unity. Error bars correspond to the SD of triplicate measurements. Significance between unirradiated controls for each triplicate data set (asterisks above individual bars) and between parental and NS subcultures (asterisks above brackets) are shown.

The capability of cancer cells to adapt their metabolic machinery to differentially utilize glycolysis or oxidative phosphorylation (OXYPHOS) in response to an evolving tumor microenvironment imparts distinct survival advantages. To determine if/how cellular bioenergetics of paired syngeneic glioma lines might change in response to acute irradiation, we subjected parental and NS cells to flux analysis to quantify oxygen consumption rate (OCR) and extracellular acidification rate (ECAR). In the absence of irradiation, basal metabolic profiles were very different between parental and NS cells. Both U251 and A172 parental cells exhibited significantly (*P* <0.005) higher OCR and significantly (*P* <0.005) lower ECAR compared to their corresponding NS cells (Figure 4, open bars).

For each pair of cell lines studies, significant changes in OCR or ECAR were not found 1 day following irradiation, with the exception of a significant 1.5-fold increase of OCR in the NS subculture of A172 (Figure 4). However, analysis of cellular bioenergetics studied 7 days after irradiation revealed several significant changes in OCR and ECAR between the paired cell lines. One week following a dose of 2 Gy, significant reduction in OCR was found for U251 and its NS subculture (U251-NS) (Figure 4). While A172 showed an increase in OCR at 1 week after irradiation, the corresponding NS subculture (A172-NS) exhibited a significant (*P* < 0.005) 2-fold drop in OCR (Figure 4, left panel). For each cell pair, irradiation significantly reduced OCR (*P* < 0.005) and more extensively 1 week after exposure in the NS subcultures (Figure 4, left panels). At the protracted post-irradiation interval of 7 days, ECAR significantly increased (*P* < 0.005) for each of the parental cultures (U251 and A172) (Figure 4, right panel). However, the opposite was found for each of the NS cells from U251 and A172, where at day 7 after irradiation, ECAR significantly dropped (*P* < 0.005) by 43% and 83% compared to sham-irradiated controls (Figure 4, right panel). Similarly, in comparison to irradiated parental cultures at day 7, ECAR levels significantly dropped (*P* < 0.05, U251 pair; *P* < 0.005, A172 pair) by 33% and 75% for NS subcultures of U251 and A172, respectively (Figure 4, right panel). For each triplicate data set (*i.e.* control, 2 Gy day1, 2 Gy day7) significance compared to the unirradiated controls is indicated (asterisks above each bar) along with significance between parental and NS subcultures (asterisks above brackets).

## DISCUSSION

The overall results of this study highlight how paired subpopulations of glioma cells exhibit differential responses to exposure to ionizing radiation. These distinct subpopulations were derived from the same glioma cell lines (U251 and A172) and were isolated and expanded under well-defined culture conditions. The resultant subpopulations have been shown to exhibit different tumorigenic behavior, where parental and SA subcultures behaved more similarly to bulk tumor cells, while NS subcultures exhibited properties more typical of invasive stem-like tumor initiating cells (STIC) described in our prior study [6]. To expand on our previous observations and gain mechanistic insight into potentially novel radiotherapeutic interventions for glioma, we initiated the present study to more completely define the radiation response of these paired subpopulations of glioma cells.

Initial work was geared toward characterizing basal growth characteristics of the paired cell lines, where both SA parental cultures and NS subcultures of U251 and A172 showed common growth capability in soft agar in the presence of serum, but markedly different growth when serum was supplemented with growth factors (Figure 1C). These growth disparities correlated with alterations in morphology (Figure 1B), while differentiation of NS subcultures stimulated the expected loss of multipotency in favor lineage restricted markers (Figure 1D). A direct comparison of SA-parental versus NS subcultures revealed differential radiosensitivity between these paired cell lines. At clinically relevant doses, survival of both paired cell lines was reduced significantly, while the NS subcultures from each cell line exhibited greater radiosensitvity (Figure 2).

Significant past work from us and others has shown that gene expression changes clearly underlie glioma heterogeneity, which can be promoted through the inter-conversion of subpopulations driven by chromosome number variation [6, 7]. Gene expression changes are also driven by the mammalian DNA damage response that regulates cell cycle progression in conjunction with cellular repair processes to minimize the impact of genotoxic insults on the integrity of the genome and cell survival. Radiation-induced oxidative stress has been shown to alter cell survival parameters through metabolic disruptions that alter the redox state and basal homeostasis of cells. Past work and current results show the capability of acute radiation exposure to elicit an acute and protracted oxidative stress in a variety of neural cell types. Temporal kinetics of radiation-induced oxidative stress in neural stem and progenitor cells show similarities with that found in irradiated subcultures of paired glioma cells. While both SA and NS subcultures exhibited elevated ROS and RNS, effects were more robust at the latter post-irradiation time (7 days) and in the NS subcultures of each cell line (Figure 3). Persistent oxidative stress following irradiation was associated with elevated radiosensitivity in the NS subcultures, suggesting that interventions targeted to redox sensitive pathways might enhance the therapeutic efficacy of glioma radiotherapy.

To determine if/how prolonged oxidative stress in irradiated cultures might impact cellular bioenergetics, flux analysis was undertaken to assess mitochondrial metabolic parameters. Analysis of un-irradiated paired cell lines indicated that the NS subcultures of U251 and A172 exhibited lower OCR and higher ECAR, consistent with reduced dependence on OXYPHOS and higher reliance on glycolysis compared to adherent subcultures (Figure 4, open bars). Shortly after irradiation (1 day), relatively minor changes in mitochondrial metabolism were observed, regardless of cell line or subculture type (Figure 4, middle bars). This was in marked contrast to the significant changes in cellular bioenergetics observed 1 week following irradiation. Adherent subcultures showed increased glycolysis (elevated ECAR) with opposing trends in OCR, indicating that irradiation had a long-term impact on the metabolic state of these cell lines (Figure 4, black bars). More striking however was the significant reduction in OCR and ECAR levels in NS subcultures, indicating that both OXYPHOS and glycolysis were compromised significantly 1 week following a dose of 2 Gy. Reduced cellular bioenergetics in NS subcultures corresponded with enhanced radiosensitivity and elevated oxidative stress, and in particular for the NS subculture of A172, that showed significant elevations in mitochondrial superoxide 1 week following irradiation.

A similarly designed study by Vlashi et al. [16], compared monolayer (similar to our SA subcultures) and 3D cultures of neurospheres (similar to our NS subcultures) of glioma cell line U87 (originally maintained in SA conditions) and glioma primary cultures (GBM-146 and GBM-176) for metabolic difference by flux analysis. They also identified differences in cellular bioenergetics between the subpopulations of glioma cells analyzed, where neurospheres exhibited a reduced glycolytic state compared to more differentiated monolayer cells. While variations between culture conditions complicate direct comparisons of bioenergetic data derived from different heterogeneous glioma samples, consistent trends point to the dynamic nature of glioma cell metabolism. Heterogeneity among gliomas complicates the identification of common trends useful for the clinic, however, renewable neurosphere formation in cultured gliomas has been shown to predict for more rapid tumor progression and poor clinical outcome [26]. Nonetheless, an other report showed that long-term cultures of neurospheres (enriched for cancer stem cells) derived from 47 human adult brain malignant tumors were not found to be predictive of tumor initiation or temozolomide resistance [27].

Past studies analyzing glioma subpopulations has found that various tumorigenic phenotypes and treatment resistance were associated with increased numbers of glioma stem-like cells expressing the CD133 cell surface marker [20, 23]. Much of this data was derived from non-adherent spheroid cultures previously enriched for CD133 positive cells [20] while other data has since identified other cell surface signatures linked to elevated growth and expression of stem cell markers [23]. Our present and past data have shown that CD133 expression is low to undetectable in adherent and non-adherent glioma subpopulations, suggesting that the significant differences in radiosensitivity and cellular bioenergetics reported here are independent of this marker. While most gliomas contain small percentages of CD133 positive cells associated with adverse outcomes [23, 24], CD133 is not always a reliable marker of glioma stem cells [28]. The expression of CD133 in human neural stem cells has been found to be cell cycle dependent [29]. The idea that CD133 marks a transient state of neural stem cells and glioma stem-like cells is supported further by findings showing that CD133 negative glioma cells can give rise to CD133 positive glioma cells during tumor formation [30]. Furthermore, in glioma cells subjected to hypoxic stress and containing dysfunctional mitochondria, CD133 expression was associated with improved survival [31, 32].

In summary, significant evidence now links changes in redox state and metabolic stress to altered radiosensitivity [12, 13]. The data presented here demonstrates further that different cellular subpopulations (SA vs NS) adapt to defined culture conditions by altering their bioenergetic profiles that impacts their sensitivity to radiation. In NS cultures, increased levels of persistent oxidative stress may enhance radiosensitivity by increasing the burden of damage in cells that can interfere with replication fidelity and promote mitotic catastrophe. Data derived from flux analysis shows that more radioresistant cells exhibit higher metabolic activities (both OXYPHOS and glycolysis) compared to more radiosensitive subcultures. These data also showed the capability of radioresistant subpopulations to switch their metabolic preference from OXYPHOS to glycolysis over protracted post-irradiation intervals. Data also demonstrates the metabolic plasticity of heterogeneous glioma cells that are capable of adapting their bioenergetic profiles in response to exogenous stress that ultimately impacts their survival response. Consequently, strategies designed to perturb the metabolism of glioma cells may provide novel therapeutic interventions for the treatment of glioma.

## MATERIAL AND METHODS

### Cell culture and irradiation

The human glioma cell lines (A172 and U251) were obtained from Alfred Yung lab in Department of Neuro-Oncology, the University of Texas M.D. Anderson Cancer Center. Neural sphere (NS) subcultures were established in Yi-Hong Zhou lab in Department of Neurosurgery, UC Irvine. All cells used in this study were subjected to 7-STR markers authentication described in Hu et al. [6]. All cells were cultured in 37°C humidified CO2 (5%) incubators before and after IR. For the glioma subpopulations described here, parental cultures were subcloned in soft agar, from which individual colonies were subsequently picked and expanded under differential culture conditions; one is in DMEM/F12 medium supplemented with 5% bovine serum as monolayer cells adherent to plastic dishes, namely serum-adherent (SA) conditions, and the other is in DMEM/F12 medium supplemented with growth factors (20 ng/ml EGF, 20 ng/ml bFGF, and 0.3% B27) as multicellular spheres in agar (1%)-coated dishes, namely non-adherent neurosphere (NS) conditions. Thus SA- or non-adherent NS subcultures were derived from single colonies and maintained under defined culture conditions. As opposed to NS subcultures, glioma parental and SA-subcultures had similar karyotypes gene expression profiles and growth rates [6].

One day prior to irradiation (IR), parental or NS subcultures were seeded in non-coated or fibronectin (10 ug/ml)-coated plates for gene expression, redox, and flux assays. Both were mixed with soft agar (see below soft agar colony formation assay) in DMEM/F12 medium supplemented with 5% bovine serum or growth factors for survival assays. Exponentially growing cultures were either sham-irradiated or subjected to γ-irradiation (0.5 or 2 Gy) while anchored using a ^137^Cs irradiator (J.L. Shepard and Associates Mark I, CA, USA) at a dose rate of 2.2 Gy/min. Immediately after irradiation, cells were placed back in incubators until the time of assay.

### Soft agar colony formation assay

5% SeaPlaque GTG Agarose (Lonza) in PBS was autoclaved and diluted to 0.5% with warm (42oC) DMEM/F12 medium supplemented with 5% bovine serum or growth factors (20 ng/ml EGF, 20 ng/ml bFGF, and 0.3% B27) to form bottom agar (1 ml per well, 6-well plate). Single cells were mixed with warm 0.3% soft agar (1000 cells per ml) in DMEM/F12 supplemented with 5% bovine serum or growth factors (20 ng/ml EGF, 20 ng/ml bFGF, and 0.3% B27) and added to the bottom soft agar layer. The mixture of cells and agar were quickly immobilized at 4oC for 15 min prior to incubation. At week 2 and 3, 1 ml of culture medium was gently added to each well. By week, colonies were counted under a microscope using a 4X lens.

### Radiation-induced oxidative stress analysis

Redox sensitive fluorogenic dyes were used to assess relative levels of reactive species in paired glioma lines at selected times after irradiation by FACS analysis. Exponentially growing glioma cells were treated for 1 hour at 37°C prior to flow cytometry with 5-(and 6-) chloromethyl-2,7-dichlorodihydrofluorescein diacetate (CM-H_2_DCFDA, 5mM, Invitrogen) for ROS/RNS detection. The cell permeable CM-H_2_DCFDA dye yields a quantifiable fluorescent signal upon intracellular hydrolysis and oxidation. For the detection of nitric oxide (NO) cells were treated with 4-amino-5methylamino-2’,7’-difluorescein diacetate (DAF, 5mM, Invitrogen). The reaction of NO with DAF leads to the production of a fluorescent signal that can be quantified upon excitation. Cells were also treated with Mitosox (0.5 mM, Invitrogen) for the detection of mitochondrial superoxide (SO), through the quantification of a red fluorescent signal derived from an oxidized ethidium derivative. Following dye loading, cells were harvested and analyzed using the EasyCyte flow cytometer (Millipore) along with FCS Express (De Novo Software, Los Angeles, CA). All data were averaged from at least 3 determinations and normalized to unirradiated controls set to unity for each time point and dye.

### Assessment of cellular bioenergetics through flux analysis

To measure mitochondrial function in giloma cells, we employed a Seahorse Bioscience XF24 Extracellular Flux Analyzer (Seahorse Bioscience, North Billerica, MA) following the manufacturer's protocol. Briefly, 1-2X105 cells/well were seeded in a non-coated or fibronectin-coated 24-well Seahorse XF-24 assay plate 1-2 days prior to analysis. On the day of metabolic flux analysis, cells were washed once with unbuffered DMEM/F12 (pH7.4) and incubated at 37°C in the same media in a non-CO2 incubator for 1 hr. Four baseline measurements of oxygen consumption rate (OCR) were taken before sequential injection of the following mitochondrial inhibitors and final concentration: oligomycin (1 μg/ml), FCCP (0.3 μM) and rotenone (0.1 μM). Three measurements were taken after addition of each inhibitor. OCR values were automatically calculated and recorded by the Seahorse XF-24 software. The basal respiration was calculated by averaging the four measurements of OCR before injection of inhibitors.

### Statistical analysis

T-Test (two tailed, equal variance) was used to determine statistical significance for surviving fraction, gene expression, redox and bioenergetic measurements between paired parental and NS subcultures, and between irradiated and non-irradiated cells.
